# Beige Adipose Tissue Identification and Marker Specificity—Overview

**DOI:** 10.3389/fendo.2021.599134

**Published:** 2021-03-12

**Authors:** Anna-Claire Pilkington, Henry A. Paz, Umesh D. Wankhade

**Affiliations:** ^1^ Arkansas Children’s Nutrition Center, University of Arkansas for Medical Sciences, Little Rock, AR, United States; ^2^ Department of Pediatrics, College of Medicine, University of Arkansas for Medical Sciences, Little Rock, AR, United States

**Keywords:** beige adipose tissue, brown adipose tissue, white adipose tissue, obesity, thermogenesis

## Abstract

Adipose tissue (AT) is classified based on its location, physiological and functional characteristics. Although there is a clear demarcation of anatomical and molecular features specific to white (WAT) and brown adipose tissue (BAT), the factors that uniquely differentiate beige AT (BeAT) remain to be fully elaborated. The ubiquitous presence of different types of AT and the inability to differentiate brown and beige adipocytes because of similar appearance present a challenge when classifying them one way or another. Here we will provide an overview of the latest advances in BeAT, BAT, and WAT identification based on transcript markers described in the literature. The review paper will highlight some of the difficulties these markers pose and will offer new perspectives on possible transcript-specific identification of BeAT. We hope that this will advance the understanding of the biology of different ATs. In addition, concrete strategies to distinguish different types of AT may be relevant to track the efficacy and mechanisms around interventions aimed to improve metabolic health and thwart excessive weight gain.

## Introduction

Adipose tissue (AT) is an integral component of increased weight gain and has garnered significant attention in the scientific community over the last few decades. White and brown ATs (WAT and BAT), being the two main types of AT in mammals, are the focus of most scientific studies. WAT stores excess energy as triglycerides, and BAT specializes in the dissipation of energy through the production of heat. In the last decade, a third and novel type of AT came to light. Because of appearance and location, these adipocytes have been deemed brite/beige adipocytes (BeATs). BeATs are brown-like adipocytes that reside in WAT depots. Chemical stimulation with *β*3-adrenergic agonists (CL316,243) or cold exposure is known to promote the development of BeAT.

The morphological, biochemical, and physiological characteristics of WAT and BAT are well described in the literature. White adipocytes are characterized by the presence of single lipid droplet consisting of triglycerides occupying 90% of the cell space. Mitochondria in white adipocytes are thin, elongated, and sparse in numbers. WAT is responsible for secretion of numerous hormones, growth factors, enzymes, cytokines, complement factors, and matrix proteins. These secretory products are responsible for the regulation of healthy adipose mass maintenance, food intake, energy expenditure, metabolism, immunity, and blood pressure homeostasis (1). In contrast, brown adipocytes contain triglycerides in smaller and multiple vacuoles. BAT is highly vascularized and contains a large number of mitochondria that are large, spherical, and packed with laminar cristae. The brown color of BAT is attributable to its high mitochondrial density and vascularization. BAT has been reported to promote a 10–20% increase in energy expenditure when compared to basal metabolic rate with an estimated contribution of 100 kcal/day (2). Because of its relatively high tissue-specific metabolic rate, BAT demands increased oxygen and a denser neural supply when compared to WAT. A key component that differentiates BAT from WAT is BAT’s physiological function of thermogenesis, which is aided mainly by the unique protein, uncoupling protein 1 (Ucp1) present in densely packed mitochondria in BAT. Stimulation by norepinephrine triggers a signaling cascade that activates Ucp1, which then uncouples aerobic respiration by dissipating the inter-membrane proton-motive force, creating a futile cycling of ions, thus generating a heat instead of ATPs (3). BeATs display characteristics of both brown and white fat cells and typically develop within subcutaneous WAT from a distinct subset of preadipocytes (4) or *via* the trans-differentiation of existing white adipocytes (5, 6). Beige adipocytes were originally observed to arise in response to cold exposure in rodents (7–9); however, studies have since identified that diet (10), exercise (11), pre-and probiotics (12), pharmaceutical agents, numerous plant-based bioactives, and even adipokines (13, 14), can also induce “beigeing” or “browning” of WAT. These regulators of BeAT are discussed in greater detail below.

BAT mass is very scarce in most adult humans (less than 1% of total body weight). Under conditions such as cold exposure, BAT can be activated, but even under these conditions, the amount of BAT remains small relative to WAT. Nevertheless, the BAT has the potential to significantly impact whole-body glucose homeostasis. This makes it appealing to consider if beige adipocytes can also play an important role in overall metabolism, even if BeAT abundance is small relative to WAT.

As alluded above, browning has been shown to occur with a variety of different stimulants. Diet has the potential to play a role in the beigeing process as different compounds, including capsinoids, polyphenolic compounds, green tea (specifically catechins), and fish oil, have been shown as promising but still need to undergo more extensive investigation (7). Certain plant bioactives, such as curcumin, may also contribute to beigeing (15). Exercise has been shown to promote beigeing by increasing the number of mitochondria in WAT and activating brown adipocyte genes, specifically Ucp1 (8). This phenomenon has been studied extensively in mice, where exercise consists of the mouse running on a treadmill. The microbiota has been of a growing interest over the years, and with its many implications into human health, it is not surprising that it may play a role in the browning process. Boosting the microbiota with pre- and probiotics, the host has demonstrated increased thermogenic capacity and therefore, an increase in energy expenditure of both BAT and subcutaneous WAT, indicative of browning assisted improved metabolism (9). Multiple pharmacological approaches have been studied to determine their efficacy in the browning process (16). These include Ppar agonists, SIRT-1, *β*-adrenergic receptor stimulation, thyroid hormone, Fgf21, and irisin (17). Each of these methods or compounds have been studied mainly in a mouse model so further research is needed to confirm the effectiveness of each strategy in the browning of human WAT and its clinical implications.

BAT is mostly located in neck and subscapular region whereas WAT is spread out in several parts of the body. Epicardial fat or fat layer underlying major blood vessels in the thoracic region tends to express BAT specific genes such Ucp1, Pgc1a, Prdm16, *etc* at high level giving it beige/bat like appearance (18–20). Beige adipocytes can be recruited or induced in WAT depots, leading to difficulties when trying to identify the exact type of AT that is present. In an attempt to overcome this challenge, multiple studies have been conducted to identify specific markers that allow for the characterization of the tissue present. Although we have come far in identifying molecular underpinnings of beige adipocyte formation in the last decade, the transcript marker identity of beige cells is still under question. Because of its similarity in appearance, location, and dubiousness of its origin, there are still no concrete measures to distinguish beige cells from classical brown fat cells.

Decades of research has provided us with a good outline to identify different adipose cell types. As lineage-tracing studies are impossible to perform in humans, there is a need for marker genes that allow distinction to be made between different classes of (human) adipocytes and adipose tissues. Such markers can thus be obtained only from animal studies and can be used to influence the development of strategies to promote recruitment of the tissues and activation of energy expenditure. This article therefore has two aims. The first is to provide an overview of transcript markers of identification laid out in literature to identify BAT, WAT, and BeAT. The second is to highlight some of the difficulties these markers pose and offer an opinion on possible transcript-specific identification of BeAT.

## Origins of Adipose Tissues

In mammals, AT forms *in utero*, in the peripartum period, and throughout life. New adipocytes are generated continually in healthy and disease stages as physiological need arises (21). AT is composed of adipose stem cells that give rise to new adipocytes and immune cells. AT is located in several parts of the body and gets its name based on its location—in rodents, examples include inguinal, interscapular, perigonadal, retroperitoneal, and mesenteric depots. These various depots develop at specific and distinguishable pre- and postnatal times, and they have discrete, distinct morphologies (22, 23). While there is a long-standing belief that WAT is derived from the mesoderm germ layer, some studies indicate that adipose depots originate from neural crest cells which derived from the ectoderm (24). And in mice, recent study had indicated that neural crest cells differentiate into both white and brown adipocytes (25). Multiple intrinsic factors play key roles in WAT formation, with distinct developmental cues and regulators determining adipose maturation and subsequent location. Pparγ and Cebp*α* have been deemed as key genetic components of WAT development during embryonic stages (26).

Brown adipocytes are derived from the dermomyotome, an engrailed 1 (EN1) (27) and myogenic factor 5 (myf5) positive cell lineage (28), which is the same lineage as that of myocytes. During the neonatal period in humans, BAT is commonly located in the dorsal region between the scapulae (29). In rodents such as rats and mice, BAT is located primarily in the back region. Despite differences in its reported origin, there are recent studies which demonstrate that a subset of white adipocytes also arises from myf5-positive cells (30), sharing this commonality with brown progenitor cells. Platelet-derived growth factor receptor alpha (PDGFR*α*)-positive lineage traces to BAT and WAT, and PDGFR*α* is present in proliferating brown and white adipose progenitors (31). Recently Oguri et al. reported expression of Pdgfr*α*, Sca1, and Cd81 on the surface of beige adipocyte progenitors (32). It is also known that there is some overlap in the transcriptional machinery utilized in the development of both WAT and BAT.

Within the last two decades, the discovery of and research focusing on beige adipocytes has taken off. In the early 2000s, Himms-Hagen et al. demonstrated the existence of brown-like adipocytes in WAT of rats treated with *β*-adrenergic agonist CL316,243 (5). Regardless of the morphological similarities between beige adipocytes and BAT, beige adipocytes originate from distinct precursor populations (4, 28). Beige adipocytes mostly emerge from a myf5 negative cell lineage, one that is similar to that of WAT. Specific groups of beige adipocytes seem to emerge from a smooth muscle-like precursor, driven by Myh11 (33). The variety of lineages for beige adipocytes leads to the conclusion that they stem from a heterogeneous population of cells. Beige cell recruitment and coincident expression of Ucp1 are dependent on external stimuli such as cold or other inducers such as *β*-adrenergic agonists or PPAR*γ* activators (4, 34). By contrast, brown adipocytes express relatively high amounts of Ucp1 without stimulation. Classically, two external stimuli have been used to trigger a browning response: cold temperatures and CL316,243 [*β*3-adrenergic receptor (*Adrb3*) agonists]. Although these two treatments have known to be stronger triggers of beigeing and have been used interchangeably; however, whether these two stimuli may induce beigeing differently at the cellular and molecular levels remains unclear. Jiang et al. report that cold-induced beige adipocyte formation requires *Adrb*1, not activation. *Adrb*1 activation stimulates WAT resident perivascular (*Acta2*+) cells to form cold-induced beige adipocytes (35). In contrast, *Adrb3* activation CL316,243 stimulates mature white adipocytes to convert into beige adipocytes. The authors went on demonstrating it using mature adipocyte-specific *Prdm16* deletion strategies, which showed that adipocytes are required and are predominant source to generate *Adrb3*-induced, but not cold-induced, beige adipocytes (35). Stimulated beige adipocytes can undergo UCP1-mediated uncoupled respiration (4, 33, 36), but the relative magnitude per cell *versus* brown adipocytes remains to be established.

## Selective Markers of Adipose Tissues

### White Adipose Tissue Markers

Being the largest AT in mass and having a distinct white/pink color makes it somewhat easy to identify WAT with the naked eye. Several marker genes have been classified to specifically identify WAT, including Ob (leptin), Hoxc8, and Hoxc9 (37). At the transcript level, leptin and adiponectin are other commonly used markers for WAT. Solute carrier family 7 member 10 (SLC7a10/Asc-1 neutral amino acid transporter, y+ system) is another more recently identified marker that was determined to be highly expressed in C57BL/6male mouse WAT while having low levels in BAT. It has been demonstrated to increase as mice gain white adipose mass, further supporting Asc-1 as a WAT marker (38, 39). In the same study conducted by Ussar et. al., Asc-1 had levels of expression similar to leptin. Garcia et al. observed higher expression of ASC-1 in mouse white adipocytes compared to beige adipocytes (40). This study also identified two other markers that have been associated with WAT, Serpina3k and Wdnm1-like (40). de Jong et al. analyzed Asc1 as a cell surface marker and, in sync with the other mentioned studies, found that its expression levels are higher in white than in brite and brown fat cells. However, they do acknowledge that complete exclusion of Asc1 being expressed in brite cells cannot be made based on their data alone (39).

Multiple studies have looked at anatomical groupings of AT and their subsequent markers. In subcutaneous white adipose sites, Ob and Hoxc9, along with Mpzl2, Ebf3, and Fbox31, were found to be the most prevalent genetic markers (41). In a study by Walden et al., specific WAT depot sites were analyzed and compared to the BAT and BeAT depot sites. They concluded that the Tcf21 gene had the greatest association with WAT (42). de Jong et al. further support the notion that Tcf21 mRNA expression levels are indicative of white adipocytes in both cell culture and tissue. However, when looking at adipose depots with the aim of identification based on validated markers, Tcf21 was expressed in epididymal WAT, as expected, but also in brite depots. This result is logical as BeAT is a mixture of WAT and BAT (39).

### Brown Adipose Tissue Markers

As a greater understanding of beige AT is gained, a significant overlap in the markers for BAT and beige adipocytes is seen, making it important to identify those that are unique to each type. The overlap is expected due to the physiological similarities between brown and beige adipocytes, but there are also differences, likely due to their divergent developmental patterns.

Multiple studies have identified LHX8 and ZIC1 as strong markers of BAT (41–44). These studies demonstrated that these markers serve well as indicators of brown, and not BeAT. Further evidence for the use of Zic1 as a BAT marker was found in an extensive study performed by de Jong et al. where all previously identified human adipose tissue markers were evaluated in cell lines and tissue. Zic1 mRNA was detectable only in interscapular BAT, both in tissue samples and cell cultures (39). However, when analyzing single adipose depots in a mouse model, only three of the five traditional BAT depots expressed Zic1. With further analysis, it appeared that the positional expression pattern of the gene was the explanation for this phenomenon, indicating that Zic1 expression is restricted to anterior depots. The absence seen in the two most posterior-like brown fat depots is indicative of them having different origins than classical BAT (39). This study gives evidence of Zic1 being a brown fat marker while also giving insight into the importance of understanding developmental origins. While this study provided evidence complementing other studies on the role of Zic1, it found conflicting evidence regarding Lhx8 as solely a BAT marker. The levels of Lhx8 mRNA were absent in WAT but found in nearly equal levels in beige adipocytes and BAT, indicating that Lhx8 mRNA may be a marker for either beige or BAT, not BAT alone (39). Eva1 (Mpzl2) is another genetic marker that has been proposed as a candidate for identifying BAT. Seale et al. determined that with Prdm16 stimulation, mRNA expression levels of Eva1 were significantly increased in BAT *versus* WAT (45). Wu et al. looked further into Eva1 by comparing expression levels in WAT, BAT, and beige adipocytes from mice and found a significantly higher expression in interscapular brown fat depots (4). In contrast to these studies, de Jong et al. found little to no difference in Eva1 mRNA levels between WAT, BAT, and beige adipocytes while also observing an increase in expression in all three tissues with cold exposure, indicating the possibility that Eva1 plays a role in functional adaptability to cold temperatures but not as a strong marker of BAT (39). Prex1, a possible BAT marker, is known for its role as a positive regulator of Ucp1-gene expression. Xue et al. found that Prex1 is present in brown adipocyte pre-cursor cells, indicating its importance in BAT development (46). There is little research that cites increased Prex1 expression levels in beige adipocytes so it is currently considered a unique marker for BAT.

As previously mentioned, brown and beige adipocytes arise from separate cell lines and are subsequently activated by different signals, so differentiating between the two tissue types can be made easier *via* their developmental histories. Ebf2 and BMP7 are two proteins that play pivotal roles in classical BAT development, with their expression being indicative of BAT presence (43). With increased awareness of BAT-specific markers along with the differences in developmental patterns, BAT and beige can be differentiated for the purposes of further research and the possibility of their metabolic implications.

### Beige Adipose Tissue Markers

**Figure 1:**
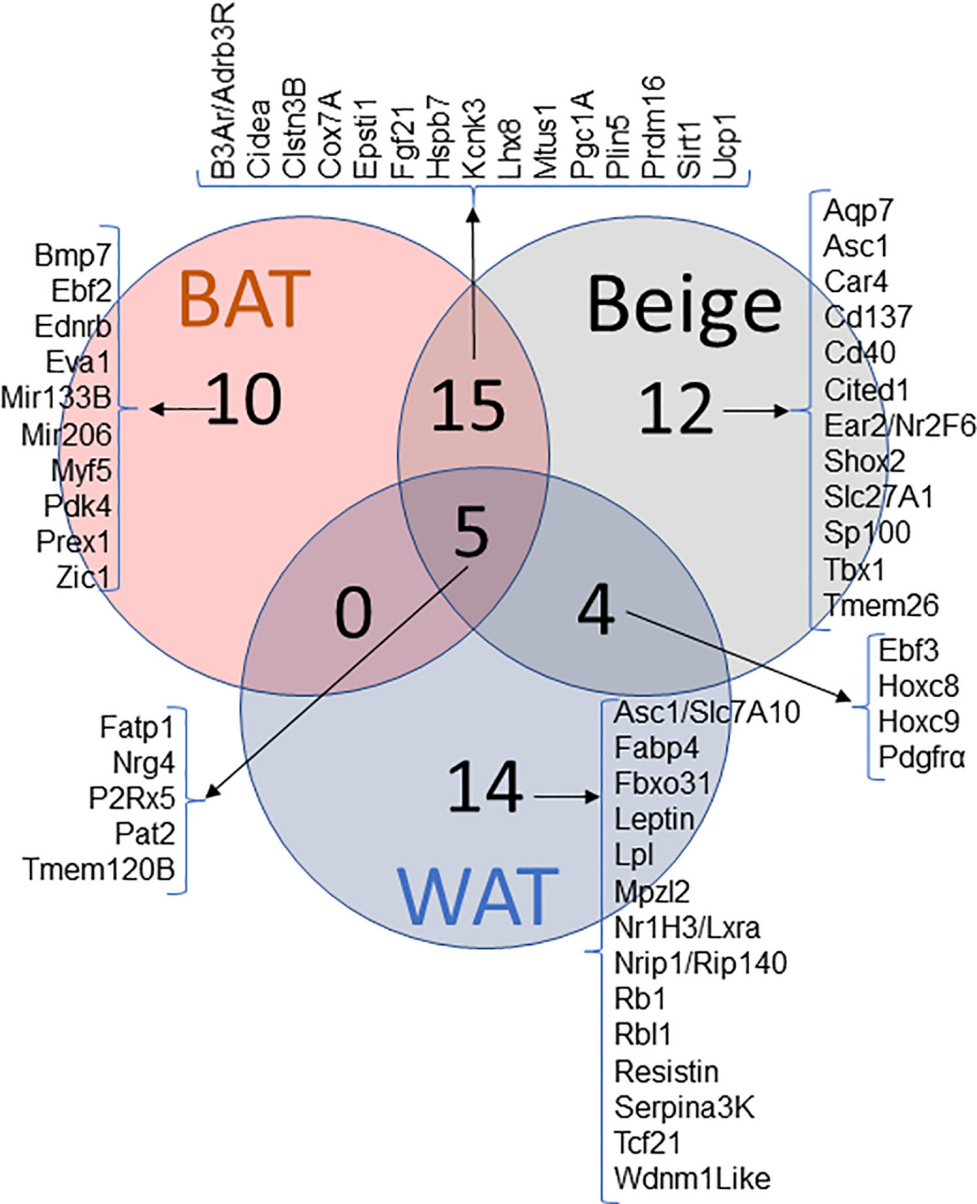
Venn diagram of WAT, BAT and Beige markers and their pattern of expression. Genes reported in literature as a marker for WAT, BAT and BeAT. Venn diagram represents the commonality and uniqueness of certain transcripts.

Identical appearance of brown and beige adipocytes makes it difficult to distinguish them one from another. In addition, similarities in physiological characteristics and marker expression make it even trickier to identify beige adipocytes. However, decade-long research of beige adipocyte development has provided some putative markers. The extensive research has allowed for the determination of markers overlapping between all three adipose tissues, along with the overlap seen between two of the types and of markers that seem to be unique to beige adipocytes. This overlap is outlined in the Venn diagram shown above with multiple of the genes of interest being discussed in this review (**Figure 1**).

### Proposed Beige Adipose Tissue Markers

*Tmem26, Tbx1, CD137, and CITED1:* Several studies have shown that CD137, Tmem26, and Tbx1 could be used as beige adipocyte markers. These mRNA transcripts and subsequent proteins are expressed at significantly higher levels in the inguinal fat depot compared to the interscapular brown fat upon *β*-adrenergic stimulation or cold exposure (4). Upon differentiation, the cells containing higher levels of these proteins also expressed higher levels of Ucp1. Cd137 and Tmem26 were also identified as cell-surface markers for native beige precursors, allowing for the isolation of these cells from fat tissues (47). Cypess et al. identified Cd137 and Tmem26 to be the best markers for beige adipocyte identification after analysis of different AT groupings (41). Liang et al. further support these conclusions as an increase in Cd137 expression was recorded in 3T3-L1 cells differentiated into beige adipocytes (48). de Jong et al. suggest that Cd137 may be able to serve as a beige adipose tissue marker at the tissue level, but not at the cellular level. However, the data collected also shows that low expression levels of Cd137 alone cannot definitively exclude a tissue from being deemed beige (39). Tbx1 and Tmem26 displayed similar expression patterns to Cd137 in the de Jong et al. study. Both genes were expressed at significantly higher levels in BeAT compared to BAT but with much smaller differences between BeAT and WAT. It is possible that Tbx1 and Tmem26 may serve as beige markers in tissues but not in cell culture (39). When analyzing Cd137, Tbx1, and Tmem26 in single adipose depots, they were found to mark brite and WAT depots, indicating that there can be brite, but non-thermogenic depots. de Jong et al. presented these markers, plus Epsti1, to be necessary for a true brite tissue (39). Epsti1 is of note here—it had been previously suggested as a brown marker but in this study was found to be at much higher levels in BeAT than BAT, leading to its consideration as a beige adipose tissue marker (39).

In contrast to the evidence presented above, in the study conducted by Ussar et al., no change in expression of either Tbx or Tmem26 was found in mice exposed to chronic cold conditions or *β*3-adrenergic stimulation (38). Garcia et al. further solidified these findings as similar expression levels of Tmem26, Tbx1, and CD137 in the beige adipocytes and white adipocytes of mice exposed to cold temperatures were observed (40). When the cells were exposed to adrenergic stimulation, CD137 increased only in white adipocytes and CITED1 increased in WAT to such a degree that it was no longer distinguishable between beige and white cell types. There is also evidence that CD137 is not only an unreliable marker of beige AT but that it actually slows down the browning process and prevents the spread of beige adipocytes (49). de Jong et al. found Cited1 mRNA to be expressed in extremely low levels in all three depots, excluding it as a marker of beige adipose tissue (39). These five markers need to be further studied to work out the discrepancies seen and to determine the relevance of these genes in accurately identifying beige AT.

### Overlapping Markers

#### Markers found in WAT, BAT and BeAT

*Pat2 and P2rx5:* Ussar et al. identified Pat2 and P2rx5 as markers of beige and brown fat (38). The study induced the browning of WAT by exposing mice to chronic cold conditions and/or *β*3-adrenergic stimulation. P2rx5 expression increased both in BAT and subcutaneous WAT after both stimuli, while Pat2 had no change with cold but demonstrated a significant increase in subcutaneous WAT with *β*3-adrenergic stimulation. These mixed results led to the conclusion that each protein may be useful in marking brown or beige adipocyte populations, as they increased in the WAT with a browning stimulus (38). Consistent with previous results, Garcia et al. showed increased P2rx5 expression in beige adipocytes compared to white adipocytes upon differentiation *via* rosiglitazone, whereas adrenergic stimulation yielded a decrease in P2rx5 expression in both beige adipose cells and white adipose cells. Pat2, on the other hand, was decreased only in white adipocytes. Finally, with cold exposure, WAT showed an increase in both P2rx5 and Pat2 (40).

The results of both studies indicate that different stimulants of WAT browning, *i.e.* rosiglitazone, chronic cold or *β*3-adrenergic stimulation, can induce the expression of these different markers. The findings regarding Pat2 and P2rx5, while useful, are also vague and will need to be further studied to determine if they have a role in specific ATs or serve as a more general adipocyte marker. Both studies demonstrate Pat2 and P2rx5 expression in BAT and BeAT, along with data supporting the markers presence in WAT.

*Nrg4:* Nrg4 has been linked to BAT activity and the browning process. A recent study conducted by Comas et al. explored the expression pattern of Nrg4 as a marker of BAT or beige AT in humans. The putative beige adipocyte marker Tmem26 showed a strong positive correlation with Nrg4 across individuals. While there are some conflicting data in regard to Nrg4’s role in thermogenesis, the observations demonstrated that Nrg4 could be a possible beige AT marker and that further studies should be conducted to confirm. Another study conducted by Christian et al. categorizes Nrg4 as a cold-induced BAT gene. They found that while BAT did have the highest levels of Nrg4 expression, subcutaneous WAT also had significant increases in Nrg4 transcript with cold exposure (50). These results suggest that Nrg4 might play a role in the browning/beigeing process. Further research needs to be done to confirm the validity of Nrg4 as a marker of a specific type of AT.

*Fgf21*: Fgf21 is another putative beige AT marker and inducer that appears to be of importance. Garcia et al. suggest that Fgf21 matches the criteria necessary to be deemed as a possible beige adipocyte marker because of its inducible nature (compared to WAT) upon treatment with rosiglitazone (40). With adrenergic stimulation, Fgf21 displayed an eight-fold greater increase in mouse beige adipose cells compared to the change seen in white adipocytes. Fgf21 was expressed in high levels in the SVF cells of the mice at room temperature and then increased further when exposed to chronic cold temperatures. Findings from this study suggest Fgf21 is involved in the dynamics of the beigeing process because it is a factor that induces beige cell recruitment in WAT while also stimulating trans-differentiation of existing adipocytes to beige cells (51).

When comparing the expression of Fgf21 in WAT, BAT, and beige adipocytes, it is higher in beige and brown cells compared to WAT, indicating Fgf21 as a hormone present during browning but that it may not be indicative of beige adipocytes specifically (52). Soundarrajan et al. studied the correlation between active BAT and various markers, including Fgf21 (53). For this study, BAT was activated when the male human subjects were exposed to cold temperatures. A positive correlation was seen between the secretion of Fgf21 and BAT activity, determined using PET/CT scans, signifying that Fgf21 may play a positive role in the physiological response to cold exposure in BAT. Additional studies have demonstrated that Fgf21 and the molecular pathway by which it could be functioning in have the capacity of inducing beige adipocyte formation. As an endocrine regulator secreted from adipocytes, Fgf21 can increase the body’s energy expenditure *via* paracrine signaling (54). Endocrine-like characteristics of Fgf21 contribute to the activation of Ucp1 and subsequently the thermogenic potential of brown and beige adipocytes (13). These properties are consistent with a connection between Fgf21 and beige function. The results also demonstrate that Fgf21 is not exclusively presented in beige adipocytes and may not serve as a marker for distinguishing between BAT and beige AT.

#### Markers Found in BAT and BeAT

There have been findings that human classical BAT is similar to mouse BeAT, leading to ambiguity in regard to identity. However, if the mouse is housed at thermoneutral conditions (30°C), such as those of humans, mouse BAT exhibits characteristics equivalent to human BAT. The seemingly small number of differential markers between BAT and BeAT can be compensated for by understanding their major differences (29). In an elegant study, Wu et al. observed that transcript signature of immortalized Ucp1+ cell line derived from inguinal AT from mouse was more similar to the expression pattern of transcripts expressed in human BAT (4). These findings were consistent with a study by Sharp et al. (39) suggesting that beige/brite cells that occur in inguinal WAT have more resemblance to classical human BAT.

*CIDEA and Ucp1:* Because of the physiological similarities between BAT and beige adipocytes, it is expected that they would have overlapping expression of CIDEA and Ucp1, two transcripts associated with the thermogenic responses of BAT. It has been proposed that these gene transcripts can be used to evaluate beigeing in mouse WAT (40). Mouse WAT exposed to chronic cold temperatures showed a significant increase in expression of both gene transcripts, with Ucp1 showing the most substantial increase. The gene transcripts do not act specifically as BAT *vs*. BeAT markers but are still valuable in terms of identifying and possibly inducing the beigeing of WAT. With the correlation seen in the increase of both Ucp1 and Cidea, it is also possible that Cidea plays a role in the thermogenicity of BeAT and BAT (39).

Ucp1 has been the focus of additional studies. It is well-known that Ucp1 is an indicator of BAT and therefore, the role that Ucp1 has in beige AT is a topic of interest. Wu et al. found that before inducing adipocyte differentiation, inguinal-derived cells from both beige and white adipose depots had low expression of Ucp1 transcript. After differentiation, with either cAMP or *β*-adrenergic agonist stimulation, the beige adipocytes had significantly increased Ucp1 expression compared to white adipocytes (4). In some cases, the Ucp1 mRNA levels in beige adipocytes were actually greater than seen in brown adipocytes. In response to high fat diet feeding Ucp1 expression tends to go up in BAT and Beige and diminishes in WAT (55). Another condition, aging is known to reduce the amount of BAT and consequently reduced Ucp1 expression (56). Thus, Ucp1 plays an important role in identifying beige AT. It may be possible that these gene transcripts will serve as better identifiers of beige AT *in vivo* compared to other proposed “beige-specific” markers.

*Pgc1α:* Pgc1*α* is known to be key in the regulation of a cascade of genes involved with energy metabolism in AT. Pgc1*α* serves as a master regulator of the thermogenic program in both BAT and beige ATs (57, 58). Mice lacking Pgc1*α* could still produce brown fat, but the thermogenic effects were diminished, indicating the indispensable role that Pgc1*α* plays in the physiological roles of BAT. Tiraby et al. demonstrated that when WAT has activated Pgc1*α*, as induced by rosiglitazone treatment, the WAT began to show characteristics of BAT, indicative of the browning process and the recruitment/trans-differentiating of beige adipocytes (59). Although several studies implicate Pgc1*α* as an important player in browning/beigeing, there is no distinction in the expression pattern in beige *versus* brown fat, making it a non-specific marker for BeAT identification.

*Prdm16:* Prdm16 has been identified as a transcriptional activator of the brown-fat selective gene program and also repressor of the WAT-specific gene program (42, 45, 60). For both BAT and beige adipocyte development, research has shown that Prdm16 serves as a key transcriptional co-regulator and is needed for development. Prdm16 appears to be an inducer of BeAT differentiation and essential in BAT, but may not serve as a valid marker for AT that is already present.

*Car4:* In a comprehensive study of several possible beige adipocyte markers, Car4 came out as one of the most convincing (40). During beige adipocyte differentiation using rosiglitazone, there was a 23-fold increase of Car4 mRNA expression (30). Interestingly, with *β*-adrenergic stimulation of both white and beige adipocytes, Car4 expression levels increased in only white adipocytes suggesting formation of beige adipocytes upon *β*-adrenergic stimulation. Car4 showed a significant increase in the stromal vascular cell (SVC) fraction of WAT derived from mice exposed to chronic cold temperatures, suggesting the abundance of transcript increased due to beigeing (30). Jong et al., in support with the results from previous studies, demonstrated a significant increase in expression of Car4 after treatment with rosiglitazone in beige adipose cultures, but this increase was seen in BAT as well, possibly eliminating the exclusivity of Car4 being a marker of beige adipocytes (52). It is also of note that warm-acclimated mice in their study had the highest expression levels of Car4 in BAT (39). When analyzing adipose depots, Car4 was found to be expressed in similar levels in all adipose depots (39). These studies provide a wide array of information regarding Car4 and the possibility of it being identified as a beige adipocyte marker. Due to the mRNA expression seen in BAT and beige adipose tissue, specifically with rosiglitazone treatment, Car4 is a marker of interest for future studies to explore the possibility of its functional role in adipose development.

#### Markers Found in WAT and BeAT

*Hoxc8 and Hoxc9:* Overlap of markers between BeAT and WAT is expected because, as with BAT, beige adipocytes do have characteristics of white adipocytes. de Jong et al. report both Hoxc8 and Hoxc9 to have similar expression levels in both tissue and cultures of white and beige fat, with near absence from brown fat (39). However, when looking at single adipose depots, both Hoxc8 and Hoxc9 were expressed through the different depots. It is of note that Hoxc8 displayed a negative correlation with Ucp1 expression (39).

*Aqp5, Aqp7, and Aqp9:* The role of aquaglyceroporins in white AT differentiation has been well-studied, and it is known that Aqp7 is especially important for WAT. In a study conducted by Silva et al., aquaporin expression patterns were noted throughout beige adipocyte differentiation and were compared to other biomarkers to determine if they could be useful in denoting beige adipocytes (61). They determined that Aqp7 and Aqp9 mRNA expression levels were upregulated throughout the beigeing process, possibly indicating these proteins as markers of beige differentiation. In differentiated beige adipocytes, expression levels of Aqp7 and Aqp5 led to the conclusion that they are the most prominent aquaglyceroporins in beige adipocytes. It is interesting that Aqp7 was expressed at significantly lower levels in beige adipocytes compared to white adipocytes, leading to the possibility that Aqp7, while important in the beigeing process, may serve as a way of comparing WAT to beige adipocytes *via* level of expression.

## Conclusion and Relevance

In conclusion, there are multiple markers that can possibly be used to identify the presence of BeAT. As made clear throughout this review, there is still conflicting evidence on the use of many of the genetic markers discussed. We now know that BeAT is derived from WAT but has similar properties to BAT, yet the full suite of regulators of BeAT and its metabolic properties, need to be better understood. The studies of BeAT have shown that cold temperature exposure and adrenergic stimulation are able to induce the beigeing of WAT in mice, and as the WAT is beiged, the presence of potential BeAT and BAT markers are increased. Expanding our current knowledge on the dynamics and identity of these factors is crucial for understanding the mechanisms that drive induction of BeAT and for considering nutritional, exercise, or pharmaceutical interventions that activate BeAT in order to improve metabolic health.

To make the best use of knowledge we gathered from previous studies, we can say two things for certain, 1) Beige cells reside and are recruited in typically subcutaneous WAT; using novel transcript markers such as Tmem26, Tbx1, CD137, or Car4 to identify BeAT is the best possible available strategy. 2) Because of the peculiar nature of recruiting sites/depots of BeAT, negative selection criteria such as expression of the above mentioned genes and in addition some of the BAT specific genes *e.g.* Ucp1, Cidea, and/or Pgc1a expression can also be used to classify the type of AT. *In vivo* imaging identifying the structural differences in adipocytes such as size of cells and number of lipid droplets in the cell can be used to aid the identification of BeAT.

Difficulties in using specific transcript expression solely has its own limitations. BeAT recruitment and expression pattern of these cells can be affected by physiological conditions (diet, age, and ambient temperature). Expression of BeAT in standard mouse models (chow‐fed, young, and at 20°C) is different from those of humans. Ongoing studies from our lab are understanding the expression pattern of specific transcript under these certain conditions.

## Author Contributions

A-CP searched the literature and wrote the manuscript. UW prepared the figures and edited the manuscript. HP, revised the manuscript. All authors contributed to the article and approved the submitted version.

## Conflict of Interest

The authors declare that the research was conducted in the absence of any commercial or financial relationships that could be construed as a potential conflict of interest.
